# Supplementary material for: Overlapping cell population expression profiling and regulatory inference in C. elegans
Source: BMC Genomics. 2016 Feb 29;17:159. doi: 10.1186/s12864-016-2482-z (PMC4772325; doi:10.1186/s12864-016-2482-z)
Supplement: Additional file 13: — Web supplement. (DOC 21 kb) [file 12864_2016_2482_MOESM13_ESM.zip › sortWeb/clusters/hier.300.clusters/47.html]

Cluster 47 

## Cluster 47

### Expression

| cnd-1 rep. 1 | cnd-1 rep. 2 | cnd-1 rep. 3 | pha-4 rep. 1 | pha-4 rep. 2 | pha-4 rep. 3 | ceh-27 | ceh-36 | ceh-6 | F21D5.9 | mir-57 | mls-2 | pal-1 | pros-1 | ttx-3 | unc-130 | hlh-16 | irx-1 | ceh-6 (+) hlh-16 (+) | ceh-6 (+) hlh-16 (-) | ceh-6 (-) hlh-16 (+) | cnd-1 singlets | pha-4 singlets | 0 | 60 | 120 | 150 | 180 | 240 | 330 | 390 | 420 | 480 | 540 | 570 | 600 | 630 | 660 | NAME | Functional description |
| --- | --- | --- | --- | --- | --- | --- | --- | --- | --- | --- | --- | --- | --- | --- | --- | --- | --- | --- | --- | --- | --- | --- | --- | --- | --- | --- | --- | --- | --- | --- | --- | --- | --- | --- | --- | --- | --- | --- | --- |
|  |  |  |  |  |  |  |  |  |  |  |  |  |  |  |  |  |  |  |  |  |  |  |  |  |  |  |  |  |  |  |  |  |  |  |  |  |  | Y51F10.13 |  |
|  |  |  |  |  |  |  |  |  |  |  |  |  |  |  |  |  |  |  |  |  |  |  |  |  |  |  |  |  |  |  |  |  |  |  |  |  |  | C18H7.10 |  |
|  |  |  |  |  |  |  |  |  |  |  |  |  |  |  |  |  |  |  |  |  |  |  |  |  |  |  |  |  |  |  |  |  |  |  |  |  |  | K05F1.9 |  |
|  |  |  |  |  |  |  |  |  |  |  |  |  |  |  |  |  |  |  |  |  |  |  |  |  |  |  |  |  |  |  |  |  |  |  |  |  |  | *anr-49* | Antisense Non-coding RNA |
|  |  |  |  |  |  |  |  |  |  |  |  |  |  |  |  |  |  |  |  |  |  |  |  |  |  |  |  |  |  |  |  |  |  |  |  |  |  | T05A8.8 |  |
|  |  |  |  |  |  |  |  |  |  |  |  |  |  |  |  |  |  |  |  |  |  |  |  |  |  |  |  |  |  |  |  |  |  |  |  |  |  | H23L24.2 |  |
|  |  |  |  |  |  |  |  |  |  |  |  |  |  |  |  |  |  |  |  |  |  |  |  |  |  |  |  |  |  |  |  |  |  |  |  |  |  | *hsd-1* | HydroxySteroid Dehydrogenase homolog |
|  |  |  |  |  |  |  |  |  |  |  |  |  |  |  |  |  |  |  |  |  |  |  |  |  |  |  |  |  |  |  |  |  |  |  |  |  |  | *gst-32* | Glutathione S-Transferase |
|  |  |  |  |  |  |  |  |  |  |  |  |  |  |  |  |  |  |  |  |  |  |  |  |  |  |  |  |  |  |  |  |  |  |  |  |  |  | *tsp-4* | TetraSPanin family |
|  |  |  |  |  |  |  |  |  |  |  |  |  |  |  |  |  |  |  |  |  |  |  |  |  |  |  |  |  |  |  |  |  |  |  |  |  |  | *ceh-79* | C. Elegans Homeobox |
|  |  |  |  |  |  |  |  |  |  |  |  |  |  |  |  |  |  |  |  |  |  |  |  |  |  |  |  |  |  |  |  |  |  |  |  |  |  | K05D4.9 |  |

### Phenotypes enriched

none found

### Anatomy terms enriched

none found

### GO terms enriched

none found

### Expression clusters enriched

none found

### Motifs enriched

|  |  |  |  |  |  |
| --- | --- | --- | --- | --- | --- |
| **Motif** | **Logo** | **Possible orthologs** | **Number of motifs in cluster** | **Enrichment** | **FDR corrected p** |
| pTH5737 |  | nhr-79 | 5 | 6.08 | 0.046 |

### Correlated (and anti-correlated) transcription factors

|  |  |
| --- | --- |
| **Transcription factor** | **Correlation** |
| ceh-79 | 0.91 |
| nhr-231 | 0.63 |
| nhr-164 | 0.63 |
| gmeb-3 | 0.59 |
| ccch-2 | 0.51 |
| C08G9.2 | 0.51 |
| ZC328.2 | 0.50 |
| nhr-222 | 0.48 |
| nhr-33 | 0.47 |
| fkh-2 | 0.46 |
| ceh-28 | 0.45 |
| unc-55 | 0.44 |
| cebp-1 | 0.43 |
| tbx-11 | 0.42 |
| tbx-38 | 0.42 |
| ets-5 | 0.42 |
| nhr-223 | 0.41 |
| tbx-34 | 0.41 |
| Y116A8C.19 | 0.41 |
| srt-58 | 0.40 |
| fkh-3 | 0.40 |
| Y116A8C.20 | 0.40 |
| sdz-38 | 0.40 |
| nhr-26 | 0.39 |
| nhr-77 | 0.39 |
| nhr-177 | -0.39 |
| 2L52.1 | -0.39 |
| nhr-73 | -0.40 |
| ceh-99 | -0.40 |
| lag-1 | -0.41 |
| pqm-1 | -0.41 |
| dhhc-4 | -0.42 |
| nhr-210 | -0.42 |
| nhr-165 | -0.42 |
| nhr-68 | -0.42 |
| nhr-109 | -0.43 |
| hlh-26 | -0.44 |
| nhr-110 | -0.44 |
| sma-3 | -0.44 |
| nhr-94 | -0.44 |
| npax-4 | -0.45 |
| dnj-17 | -0.45 |
| lin-22 | -0.47 |
| nhr-16 | -0.47 |
| nhr-79 | -0.49 |
| nhr-150 | -0.51 |
| nhr-127 | -0.51 |
| nhr-152 | -0.53 |
| Y41D4B.26 | -0.54 |
| nhr-175 | -0.56 |

### ChIP peaks enriched

none found
